# *De novo* assembly and annotation of *Hyalomma dromedarii* tick (Acari: Ixodidae) sialotranscriptome with regard to gender differences in gene expression

**DOI:** 10.1186/s13071-018-2874-9

**Published:** 2018-05-24

**Authors:** Chaima Bensaoud, Milton Yutaka Nishiyama, Cherif Ben Hamda, Flavio Lichtenstein, Ursula Castro de Oliveira, Fernanda Faria, Inácio Loiola Meirelles Junqueira-de-Azevedo, Kais Ghedira, Ali Bouattour, Youmna M’Ghirbi, Ana Marisa Chudzinski-Tavassi

**Affiliations:** 1 Université de Tunis El Manar, Institut Pasteur de Tunis, LR11IPT03, Service d’entomologie médicale, 1002 Tunis, Tunisie; 2 Laboratório Especial de Toxinologia Aplicada, CeTICS, Instituto Butantan, Av. Vital Brazil, 1500, CEP, São Paulo, 05503-900 Brazil; 3 Université de Tunis El Manar, Institut Pasteur de Tunis, LR11IPT09, Laboratoire de Bioinformatique, Biomathematique et biostatiqtiques, 1002 Tunis, Tunisie; 4 Laboratório de Biologia Molecular, Instituto Butantan, Av. Vital Brazil, 1500, CEP, São Paulo, 05503-900 Brazil

**Keywords:** *Hyalomma dromedarii* tick, Camels, Tunisia, Sialotranscriptome, Gene expression, Gene enrichment

## Abstract

**Background:**

Hard ticks are hematophagous ectoparasites characterized by their long-term feeding. The saliva that they secrete during their blood meal is their crucial weapon against host-defense systems including hemostasis, inflammation and immunity. The anti-hemostatic, anti-inflammatory and immune-modulatory activities carried out by tick saliva molecules warrant their pharmacological investigation. The *Hyalomma dromedarii* Koch, 1844 tick is a common parasite of camels and probably the best adapted to deserts of all hard ticks. Like other hard ticks, the salivary glands of this tick may provide a rich source of many compounds whose biological activities interact directly with host system pathways. Female *H. dromedarii* ticks feed longer than males, thereby taking in more blood. To investigate the differences in feeding behavior as reflected in salivary compounds, we performed *de novo* assembly and annotation of *H. dromedarii* sialotranscriptome paying particular attention to variations in gender gene expression.

**Results:**

The quality-filtered Illumina sequencing reads deriving from a cDNA library of salivary glands led to the assembly of 15,342 transcripts. We deduced that the secreted proteins included: metalloproteases, glycine-rich proteins, mucins, anticoagulants of the mandanin family and lipocalins, among others. Expression analysis revealed differences in the expression of transcripts between male and female *H. dromedarii* that might explain the blood-feeding strategies employed by both genders.

**Conclusions:**

The annotated sialome of *H. dromedarii* helps understand the interaction of tick-host molecules during blood-feeding and can lead to the discovery of new pharmacologically active proteins of ticks of the genus *Hyalomma*.

**Electronic supplementary material:**

The online version of this article (10.1186/s13071-018-2874-9) contains supplementary material, which is available to authorized users.

## Background

Ticks are hematophagous arthropods that injure their hosts. More dangerously, they are vectors of many pathogens including arboviruses, spotted fever *Rickettsia*, *Anaplasma*, *Borrelia*, *Babesia*, that cause human and veterinary diseases worldwide [1–5]. Even though chemical acaricides proved to be effective against tick infestations [6], the problem of tick resistance is becoming a problem and a primary cause of growing economic losses [6]. Research on alternatives to the use of acaricides is strongly focused on the development of anti-tick vaccines that are considered to be a more cost-effective, environmentally safe strategy [7].

One of the most promising strategies of the synthesis of anti-tick vaccines is based on tick salivary molecules that have immunosuppressive properties that are secreted during blood-feeding [8]. Indeed, ticks have developed an arsenal of salivary molecules including anti-hemostatic, anti-inflammatory and immunomodulatory compounds that are involved in avoiding host defense, enabling them to remain safe while taking their blood meal [9]. In addition, bioactive molecules secreted in tick saliva are involved in the transimission of the pathogens to the host: this phenomenon also called saliva-assisted transmission (SAT) [10]. As they are long-term blood-feeders, ticks are constantly threated by host defense pathways that might interrupt the blood meal and even kill the tick [9]. Although male and female ticks are both hematophagous, they have very different feeding behaviors. For example, females have a longer blood-feeding process than males, and their body weight differences are more than 50-fold after feeding [11, 12].

The first tick *Ixodes scapularis* genome became available only in 2016 [13], as tick genomes are typically large, highly repetitive, and difficult to assemble. Recent advances in tick sialotranscriptomic analysis, combined with NGS projects and functional studies, have provided the genomic datasets needed for further research and have shed light on a large number of the active molecules that could explain tick salivary gland physiology and identify vaccine candidates [14]. RNA sequencing (RNAseq) is an excellent technique for investigating several non-model organisms, such as ticks, cost-effectively [15, 16]. For last three decades, given the valuable information provided by tick sialome data analysis, many tick salivary gland transcriptomes were generated from adult males and females as well as on other tick development stages [17–23]. These transcriptomes pointed out the complexity of salivary protein families in the different tick species and identified new candidate genes involved in feeding. However, no such in depth transcriptome has yet been generated for *Hyalomma dromedarii* Koch, 1844, despite it being one of the most economically important ticks especially in the Saharan regions.

*Hyalomma dromedarii* is closely associated with camels, which are the main hosts of the adult ticks [24]; nymphs and larvae are more ubiquitous and can parasitize rodents, hedgehogs and birds [25]. The species represents nearly 90% of ticks infesting camels and is distributed wherever camels are present, in southern Russia, the Far, Middle, and Near East, North Africa and south of the great northern deserts as far south as Somaliland and northeastern Kenya [24]. *Hyalomma dromedarii* is the vector of the life-threatening Crimean-Congo hemorrhagic fever virus [26]. Its association with camels has an economic importance as the health and reproduction of camels are affected by heavy tick infestations [27]. The long-term blood meal of *H. dromedarii* adult ticks implies the involvement of a large, diverse number of salivary gland components. However, to our knowledge, no report has described these important molecules in *H. dromedarii*. In the present work, we have therefore aimed to: (i) *de novo* assemble the sialotranscriptome of *H. dromedarii* that enriches sequences information available in gene databases; (ii) provide a high-quality annotation and characterization of tick secretory proteins; and (iii) specify genes putatively associated with tick blood meal by exploring the differential expression between tick genders.

## Methods

### Ticks and salivary gland collection

*Hyalomma dromedarii* ticks were collected from camels in the Saharan bioclimatic zone of southern Tunisia (33°25'908"N, 009°00'952"E). The camels were thoroughly inspected; especially the inguinal region and the legs, preferential attachment sites of this species. Partially engorged ticks, at different nearly feeding stages, were removed manually from the camel body, placed in flasks containing a piece of filter paper and then brought to the laboratory. Each tick was identified using a taxonomic key [28]. Within the first hour of collection, ticks were washed and fixed in paraffin by their legs and then lateral cuts were made with a scalpel to remove the scutum. Salivary glands (SG) were teased away from other organs using ultra fine forceps. SG were washed in ice-cold PBS, pH 7.2 and stored at -80 °C until RNA isolation. Salivary glands were pooled by sex, resulting in one sample for female and one sample for male ticks.

### mRNA isolation, quantification and integrity

Total RNA was extracted from 50 pairs of *H. dromedarii* SG, of each gender, using Trizol total RNA extraction reagent (Ambion, Life Technologies, CA, USA). SG were homogenized and the total RNA was extracted and re-suspended in RNAase-free water according to the manufacturer's protocol. RNA integrity was assessed using 2100 Bioanalyzer (Agilent Technologies, Santa Clara, CA, USA). The mRNA was prepared with magnetic beads with an oligo (dT) according to Dynabeads® mRNA DIRECT kit (Ambion, Life Technologies, CA, USA). mRNA was quantified by Quant-iT™ RiboGreen® RNA reagent and Kit (Invitrogen, Life Technologies Corp., Carlsbad , USA). The integrity of mRNA was evaluated in a 2100 Bioanalyzer (Agilent Technologies, CA, USA).

### cDNA library preparation, sequencing and pre-processing analysis

Extracted mRNA was further processed for cDNA libraries constructions following the stranded TruSeq RNA Sample Prep Kit protocol (Illumina, San Diego, CA, USA). Briefly, selected poly-A-RNA was fragmented and primed with random hexamers. Fragmented RNA was reverse transcribed in order to generate first strand cDNA. Indexing adapters were ligated to cDNA for hybridization onto the flow cell of Illumina HiSeq 1500 sequencing machine. The size distribution of the cDNA libraries was measured by 2100 Bioanalyzer with DNA1000 assay (Agilent Technologies, CA, USA). An ABI Step One Plus Real-Time PCR System were used in quantification of the sample library before sequencing. The cDNA libraries were sequenced on the Illumina HiSeq 1500 System, in Rapid run mode, generating 2 × 150 bp paired-end reads, according to the standard manufacturer protocol.

### Pre-processing of RNA-Seq raw data and *de novo* transcriptome assembly

Using Illumina Casava software (v1.8.2), with Illumina quality control QC > Q30, two paired-end fastq files were generated corresponding to each *H. dromedarii* gender. RNA-Seq raw data reads were filtered by PhiX contaminant, using the software bowtie2 version 2.2.3 [29] and by quality, read size (> 40 bp), homopolymer (> 90%), low complexity sequences (> 90%) and poly-A/T/N tails and adapters, using the software fastq-mcf version 1.04.662 [30]. Paired-end read sequences with good quality from the two genders were assembled to obtain one sialotransciptome, using the Trinity assembler using the paired-end option, with parameters CuffFly and *in silico* normalization of reads with a maximum of 50× coverage [31]. The assembled transcripts were filtered according to their sequence length and those lowly expressed (that likely represent artifacts) by filtering with a Fragments Per Kilobase of exon per Million fragments mapped (FPKM < 1) [32]. No assumptions are made about genes with low abundance levels, but they are not easily distinguishable from background noise. The completeness of the transcriptome was also estimated by the presence of sequences belonging to the set of ultra-conserved eukaryotic proteins, tested using the CEGMA pipeline [33] and BUSCO approach [34] based on eukaryotic database.

### Functional annotation of *H. dromedarii* transcriptome

The transcriptome was annotated using two approaches, BLASTx similarity tool [35] (*e*-value < 1 × 10^-5^) to compare to the gene ontology (GO) database [36], the nucleotide sequences to the NCBI non-redundant protein database (NR), UniProt Knowledgebase translated EMBL-Bank predicted peptides from the *I. ricinus* genome, Animal Toxin Database (ATDB) [37] and search for Signal Peptide in the predicted proteins [38]. The second approach, dCAS (desktop annotation system), an automatic annotation server [39], was used to assign transcript to the Pfam [40], (SMART) [41], eukaryotic ortholog groups (KOG) [42] databases using rpsblast tool [35]. Results were mapped into an excel spreadsheet which was integrated to the first approach and is presented in Additional file 1: Table S1 as described in the dCAS software tool [39]. TransDecoder utility [43] was used to predict the Open Reading Frames (ORFs) from the assembled transcripts, and putative signal peptides were predicted using the software SignalP 4.0 [38]. The predicted amino acid sequences were BLASTp aligned against specific protein databases. A priority order of UniPro- tKB/TrEMBL, Pfam database and NR-NCBI was used for annotation and selection of best candidate for each transcript. Thus, transcripts were filtered according to the following steps: (i) blast against Uniprot-Acari with *e*-value < 0.05; (ii) blast against Pfam with *e*-value < 0.05; (iii) deletion of “unknown” and “uncharacterized” Pfam annotations; and (iv) the sum of female and male TPM must be greater than 1.

Predicted proteins were kept in the dataset if a significant BLASTp (*e*-value < 1 × 10^-5^) or domain-based match (*e*-value < 1 × 10^-3^) was obtained.

The annotation of KEGG pathways and KEGG orthology (KO) were assigned to assembled transcripts using the online KEGG Automatic Annotation Server (KAAS version and date) [44]. The Bi-directional Best Hit (BBH) method was used to obtain KEGG Orthology (KO) assignment with BHRs Score ≥ 60, and *I. scapularis* genome (version) was used as reference.

### Phylogenetic analysis

The nucleotide sequences of each individual sequence were translated into amino acid sequences using transdecoder, only the Kunitz domain region (with the 6-cysteines motif) were aligned using ClustalW [45] with default parameters. Thereafter we manually edited the amino acid sequences using Seaview [46] and performed the phylogenetic analysis with this same tool. Next we selected the best evolutionary model with ProtTest 2.4 [47]. Prottest selected the protein evolution model that best fit in the monolaris sequence alignment using default parameters: WAG with site heterogeneity model gamma þ invariant sites. Bayesian analyses were carried out using Markov chain Monte Carlo (MCMC) implemented in BEAST 1.7.5 software [48]. We ran four independent MCMC searches using distinct randomly generated starting trees. Each run consisted of 50,000,000 generations, and the trees were sampled every 1000 generations. Convergence was inspected in Tracer v1.5 [48], and uncertainties were addressed as 95% HPD intervals. All runs reached a stationary level after 10% 'burn in' with a large effective sample size. Trees obtained after the ‘burn in’ step were used to generate a maximum clade credibility tree with TreeAnnotator v1.7.5 [48], using a majority rule. The obtained tree was visualized and edited using FigTree v1.4.0 (available at http://tree.bio.ed.ac.uk/software/Figtree).

### Transcripts expression analysis

For the expression profile analysis, the assembled transcripts were filtered by putative ribosomal genes using as reference the Metazoa rRNA database [49] and BLASTn alignment with parameters of *e*-value < 1 × 10^-20^, query coverage > 60% and query identity > 70%. In order to estimate transcript abundance we aligned each set of reads back to the *H. dromedarii* assembled transcriptome and maximum likelihood abundance estimates were obtained using the RSEM method [50]. Final abundance estimates were calculated as Fragments Per Kilobase of exon per Million fragments mapped (FPKM) and Transcripts Per Million (TPM) values. We subsequently identified differentially expressed genes between the male and female tick samples with the EdgeR Bioconductor software package [51], a preferred methodology for studies lacking biological replicates, and extract those transcripts that are at least four fold differentially expressed with false discovery-corrected statistical significance of at most 0.001.

For the identification of gender-specific transcripts the sample reads of male and female were mapped individually to the transcriptome assembly and the transcripts were classified in each group (male, female, both) based on the transcripts abundance with FPKM > 1. All plots have been developed using R and *ggplot2* [52]. Statistics analysis for enzyme families and classes were done using chi-square statistics [53].

### Transcriptome enrichment analysis

The identification and categorization of metabolic pathways in *H. dromedarii* based on KEGG database and integration with the lists of differentially expressed genes in each gender was possible using “the Enrichment analysis for the metabolic pathways” based on GeneMerge software v1.4 [54]. The GeneMerge uses a hypergeometric distribution and apply a Bonferroni correction for a more appropriated and significant identification of enriched pathways. The analyses were divided into two sets, a group of 557 differentially expressed genes in Tick Female and another group of 353 differentially expressed genes in Tick Male, and a filter was applied for the identification of the most enriched pathways with corrected *P*-value ≤ 1 × 10^-3^ and FDR ≤ 1%.

## Results and discussion

### Overview of the sialotranscriptome of *Hyalomma dromedarii*

Next-generation sequencing using HiSeq 1500 Illumina technology was conducted to determine mRNA sequences of the salivary glands of male and female *H. dromedarii* collected in south Tunisia. A total of 330,285,649 paired-end reads were generated for *H. dromedarii* salivary glands having an average size of 808.74 nucleotides. Adequate adapter trimming and quality filtering discarded about 5,076,399 (2.5%) and 3,255,818 (2.4%) reads for male and female, respectively. Details of filtered RNA-Seq raw data results are shown in Table 1. Only sequencing reads longer than 80 nucleotides were used to assemble primary transcripts that were classified according to their putative functions and plotted in hyperlinked excel spreadsheets (available in Additional file 1: Table S1). The combined assembly of the sequences from male and female led to the extraction of 15,342 transcripts after PFAM filter. Such a high number of transcripts was expected, and it confirms the complexity of ixodid sialotranscriptomes already examined in other studies [17–23, 55]. Indeed, tick saliva is known for the complexity of its molecules, and in many cases, their redundancy [14].

**Table 1:** Filter of *Hyalomma dromedarii* RNA-Seq raw data results

	Female		Male	
	No. of reads	% of reads	No. of reads	% of reads
Total reads (raw data)	133,633,885	–	196,651,764	–
Total good reads (approved)	13,037,8067	97.56	191,575,365	97.42
Total filtered reads (removed)	3,255,818	2.44	5,076,399	2.58
Too short after clip	2,723,639	2.04	4,198,316	2.13
Filtered on quality	488,212	0,37	796,059	0.4
Filtered on homopolymer	39,730	0.03	75,314	0.04
Filtered on low complexity	4,237	0	6,710	0
Trimmed reads (not totally removed)	22,868,516	17.11	32,950,850	16.76

**Fig. 1:**
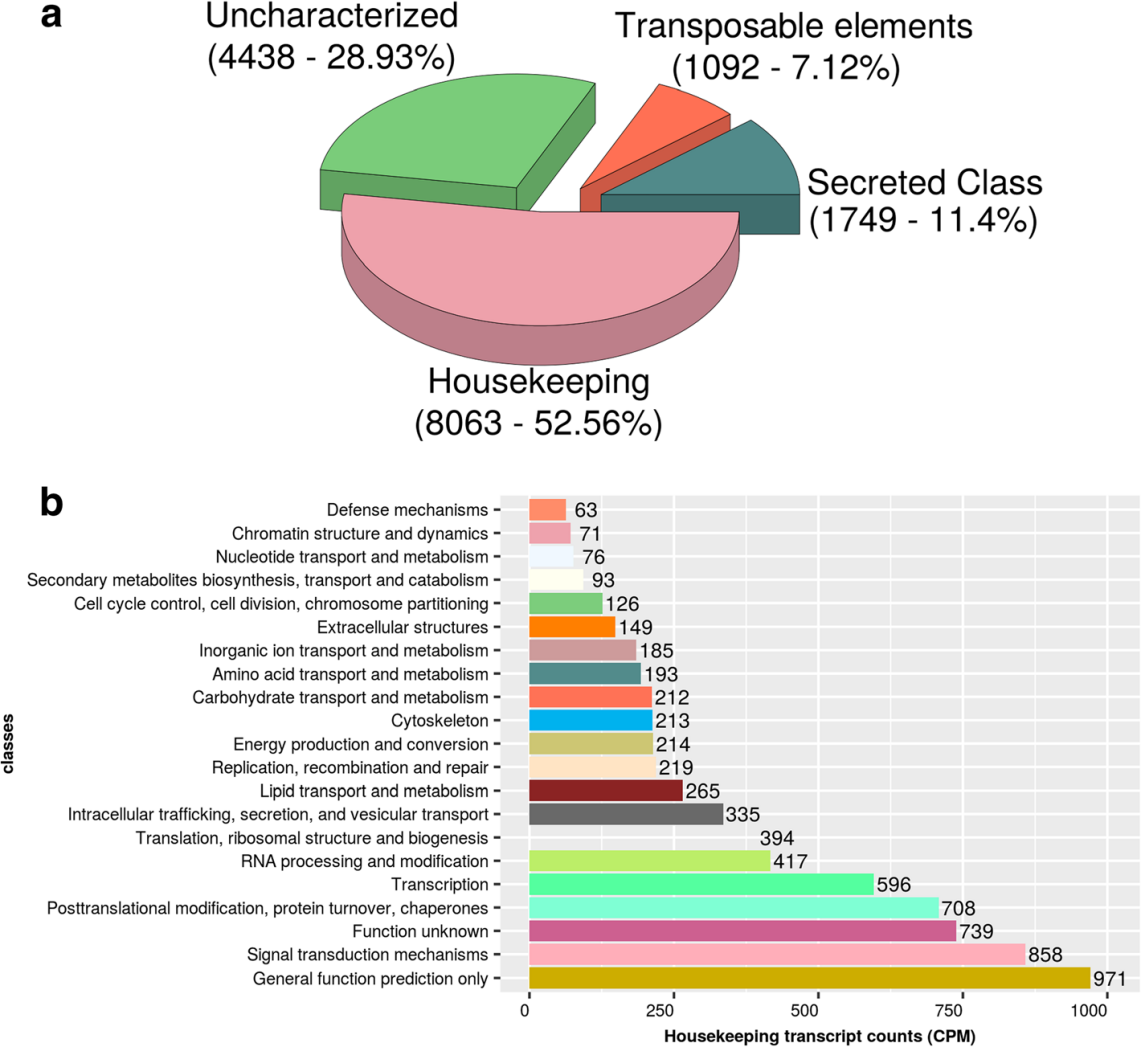
Functional classification of *Hyalomma dromedarii* tick transcripts. **a** Sialotranscriptome of *H. dromedarii* was divided into 4 categories: Housekeeping, Uncharacterized, Transposable elements and Secreted. **b** Housekeeping class of transcripts found in the sialotranscriptome of *Hyalomma dromedarii* (only families with high transcript count were represented)

For the past three decades, a reiterated transcript classification was followed for nearly all described tick sialotranscriptomes according to the function of proteins regarding tick-host interactions [18, 19, 21–23, 55, 56]. Following the same approach, the transcripts of the *H. dromedarii* were classified into 4 main categories: housekeeping, secreted, transposable elements, and transcripts with unknown function classes (Fig. 1a). A total of 1749 transcripts (11.4% of the whole transcriptome) were associated with the secreted class, while 8063 transcripts (52.56% of the whole transcriptome) belonged to the housekeeping class. Transposable elements represented almost 7.12% of all transcripts, while the rest of the reads were mapped to transcripts of unknown function (28.93%). All transcripts and their matches to several databases are available in Additional file 1: Table S1.

It is worth mentioning that the sialotranscrpitome was obtained from ticks collected on camels. These ticks might be infected by several pathogens that can affect their transcriptome. While our data need further validation using quantitative (q) RT-PCR, they do provide important information on *H. dromedarii* sialotranscriptome.

### Housekeeping class

A total of 8063 transcripts (52.56% of the total numbers of transcripts) were attributed to the housekeeping class, expressed in the SG from *H. dromedarii*. The group of housekeeping genes was divided among 156 more detailed functional subgroups of which 8033 matched the KOGG database (Table 2; Fig. 1b; Additional file 2: Table S2). The largest transcript count was attributed to the signal transduction mechanisms family, post-translational modification, protein turnover, the chaperones family, transcription and RNA processing and modification families with transcript counts of 858 (10.7% of housekeeping transcripts), 708 (8.8%) and 596 (7.4%), respectively. The presence of these major subgroups was expected as the major role of salivary glands is the secretion of the saliva. In addition, the following proteins involved in host immunity and inflammation, such as enzymes related to detoxification and oxidative metabolism, were identified: sulfotransferases, selenoproteins, superoxide dismutase and peroxidases (Additional file 2: Table S2). Similar results were reported for *Amblyomma maculatum* [57] and *Rhipicephalus pulchellus* [21]. The high amount of housekeeping/intracellular detoxification enzymes may be due to their unconventional secretion to the extracellular medium, where they play more extracellular functions that are not usually associated with their intracellular functions [58]. Furthermore, they may play different roles in the tick-host interface [59]. This distribution resembles that of previously described sialotranscriptomes [17–23].

**Table 2:** Number of transcripts, KEGG orthologs (KOs) and enzymes summarized by class

	Transcripts	KOs	Enzymes
Oxidoreductases	263	137	101
Transferases	628	365	209
Hydrolases	357	197	123
Lyases	79	50	39
Isomerases	52	32	24
Ligases	128	65	45
No Enzymes	1625	960	0

### Transposable elements

In our study, 1092 transcripts (7.12% of the sialotranscriptome) were annotated as transposable elements (TEs) (Fig. 1a) belonging to different types such as retrovirus-like element (class I) and a group of coding solely for a transposase protein with inverted terminal repeats (class II). Our results revealed the existence of both types of TE retrotransposon (gypsy, bell, outcast, Jockey, L1) and DNA transposon (PiggyBac, Mariner) (Additional file 3: Table S3). TEs are DNA sequences that can be integrated elsewhere in a genome and with few exceptions, have been identified in all eukaryotic genomes sequenced to date [60]. TEs have the potential to provide regulatory and/or protein coding sequences at a new integration site [61] and were described in nearly all published tick sialotranscriptomes.

### Secreted proteins

**Fig. 2:**
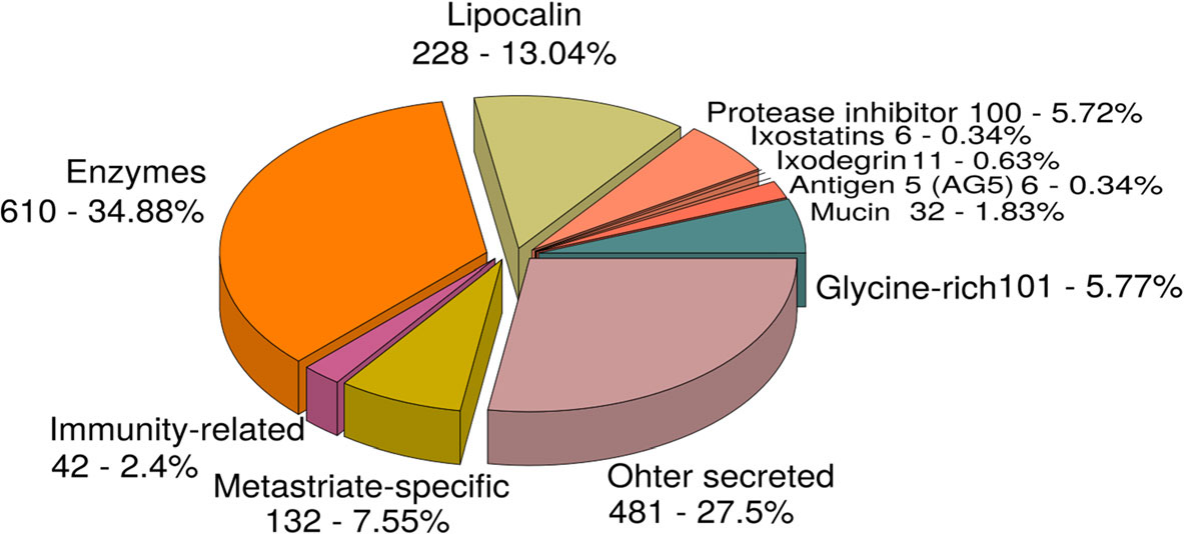
Secreted class of transcripts of the sialotranscriptome of *Hyalomma dromedarii*

In order to classify annotated transcripts to a secreted class, we referred to a previous catalog of tick proteins [62] and to the recently published tick sialomes [18, 19, 21, 22]. A total of 1749 (11.4%) transcripts were classified further into 11 families (Fig. 2) including enzymes, lipocalin, protease inhibitors, glycine-rich, metastriate specific, immunity-related, mucin, ixodegrin, ixostatin and antigen 5 and other secreted proteins. We also calculated the relative abundance of each secreted protein family in the *H. dromedarii* sialotranscriptome. All results are discussed in the following sections.

### Enzymes

**Fig. 3:**
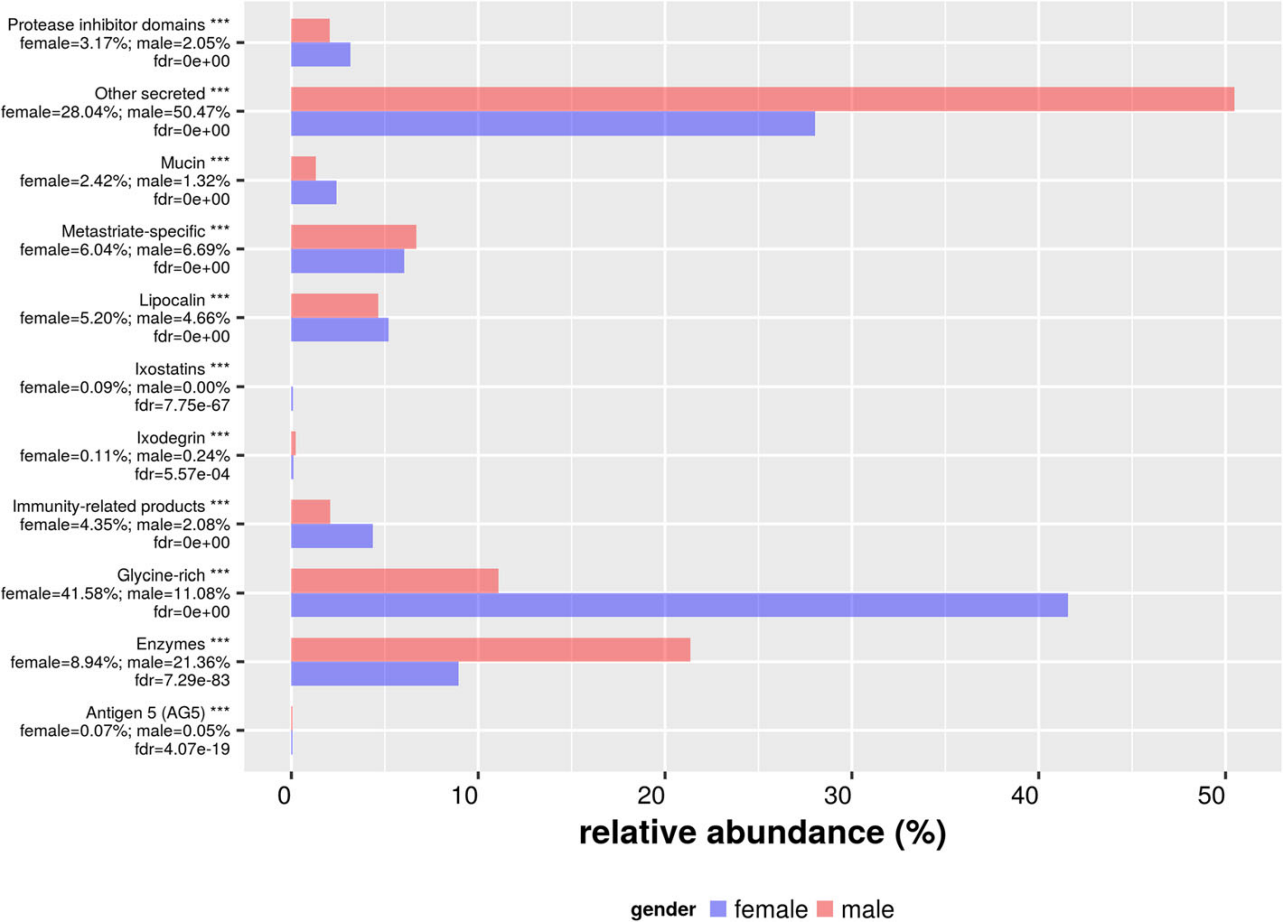
Relative abundance for each secreted protein family

Our results show that enzymes are the most abundant group in the annotated dataset of *H. dromedarii*: they total 610 (34.9%) transcripts (Fig. 2), including serine protease, metalloprotease (ADAM and M13/neprilysindomain), lipase, endonuclease, 5'-nucleotidase/apyrase, ectonucleotide pyrophosphatase/phosphodiesterase and other proteases. The relative abundance of enzymes was higher for *H. dromedarii* male SG transcriptome (Fig. 3). During blood-feeding, the mass of tick salivary glands increases due to the proteolytic activation required for countering host mechanism defenses. Indeed, proteases are powerful putative weapons of hematophagous ectoparasites [63]. Although enzymes play a very important role in the success of a tick achieving its blood meal by maintaining the feeding pool, and contribute to keeping the gut contents in liquid form [64], we can make no assumptions about whether the difference in relative abundance reflects differences in the physiological behavior of males and females. Therefore we studied gene expression for each enzyme family separately by calculating Log fold change (LFC) (log2 (TPM female/TPM male)). Relative Abundance for each secreted protein family. To compare relative abundance between females and males a chi-square test was performed given *χ*^2^ = 131810, *df* = 13, and *P* < 0.001, therefore we can infer that secreted protein profile between genders are statistically different. Details on the most important families are described below.

#### Metalloproteases

Metalloproteases are proteases requiring a metal ion, usually Zn2+, for catalysis [65]. They often have extra domains that can interact with matrix proteins. Their importance and pluripotency makes it unsurprising to find 145 of transcripts (23.8% of enzymes) related to the metalloprotease family compared to other enzymes found in the sialotranscriptome of *H. dromedarii*. Transcripts of this family were expressed in both male and female ticks. While only 64 transcripts were overexpressed in *H. dromedarii* male SG, of which 41 had significant LFC, 81 transcripts were highly expressed in female ticks, of which 54 had a significant LFC. This protein family, which is commonly found in tick saliva, salivary glands, ovaries and the mid-gut, plays an important role in countering host inflammation, immunomodulation, fibrinolysis, blood protein digestion, nociception, vitellogenesis, remodeling the extracellular matrix and pathogen transmission [21, 66]. Metalloproteases were characterized in several tick species: *I. scapularis*, *A. americanum*, *Haemaphysalis longicornis*, *Rh. microplus* and *Ornithodoros savignyi* [56, 67, 68]. It has also been shown that metalloproteases are involved not only in avoiding host defense mechanisms, but also for spermiogenesis and fertilization [68, 69]. Of metalloproteases, our results showed a total of 9 transcripts from *H. dromedarii* sialotranscriptome, which was revealed to be members of the ADAM family, of which 6 were expressed exclusively in *H. dromedarii* male ticks. ADAMs, originally known as MDC proteins (metalloproteinase/disintegrin/cysteine-rich), belong to the Metzincins superfamily of metalloproteases and display a series of biological functions including inhibition of cell adhesion, migration and angiogenesis [70]. It was suggested that members of this family included a sperm surface enzyme important for normal fertility [71]. A total of 38 transcripts of M13/neprilysin were identified in *H. dromedarii* sialotranscriptome; 20 transcripts were overexpressed in female ticks and 18 in male ticks, of which only 14 had significant LFC. Recent studies have highlighted the involvement of this family in engorgement, tick egg hatching success, and changes in their 16S-rRNA-based microbial loads [72]. These could function by destroying inflammatory peptidic mediators such as cytokines, anaphylatoxins or bradykinin from the hosts [21].

#### Endonucleases

**Fig. 4:**
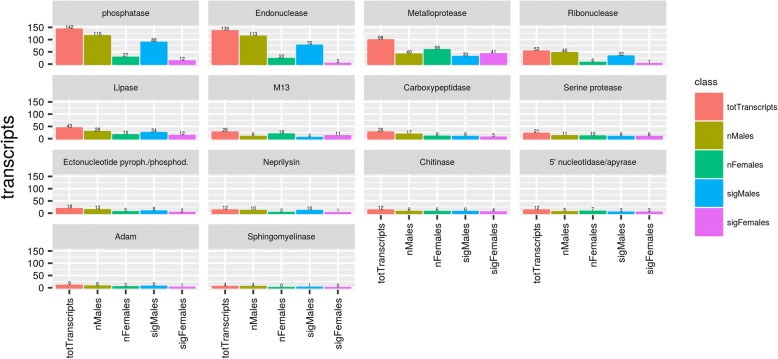
Differentially expressed enzyme families in *H. dromedarii* male and female ticks

Our analysis of the *H. dromedarii* sialotranscriptome revealed 135 transcripts that were assigned to endonucleases, which expressed significantly more in male (76 *vs* 2) than female salivary glands (Fig. 4). Endonucleases in *H. dromedarii* might play an important role in destroying neutrophils extracellular traps (NET) and therefore in enhancing parasite infectivity as reported in a previous study [73]. Expression of such enzymes by salivary glands enhances the host-parasite interaction and could be lysosomal or have another housekeeping function [62, 74]. Secreted endonucleases were identified, for the first time, in the mosquito *Culex quinquefasciatus* salivary glands and proven to play a role in blood-feeding by diffusing pharmacologic components through the host dermis that lowers the viscosity of the lacerated skin matrix [75].

#### Serine proteases

Twenty-one transcripts of serine proteases were found in *H. dromedarii* sialotranscriptome after our analysis (Fig. 4). Eleven transcripts were overexpressed in male *H. dromedarii* SG, of which 8 have statistically significant LFC while 10 were expressed exclusively in female SG, of which 8 have statistically significant LFC. Serine protease overexpression in males has been previously reported and these differences have been associated with tick reproductive biology. In fact, males have specific seminal fluid serine proteases that play in important role in spermatozoid survival [76]. Serine proteases interfere in many biological processes including cytogenesis, apoptosis, angiogenesis, neuronal plasticity, zymogen processing, matrix remodeling, immune response, inflammation, blood coagulation, and fibrinolysis [77–79]. Several serine proteases that enable ticks to establish blood pools have been described, such as longistatin in *Haemaphysalis longicornis* [80]. Serine carboxypeptidase from the tick midgut can hydrolyze bovin hemoglobin supporting serine proteases role in tick feeding success [81]. Serine proteases were also identified in male ticks such as the *Rhipicephalus pulchellus* and in other arthropods such as *Drosophila* and bumblebees [22, 69, 70, 82, 83]. They may also play a role in the reproductive biology of ticks [84].

#### 5'-nucleotidase/apyrase

Twelve transcripts were annotated in *H. dromedarii* as 5'-nucleotidase/apyrases with 3 transcripts overexpressed specifically in each gender (Fig. 4). 5'-nucleotidase/apyrase are very common in the saliva of hematophagous arthropods where they hydrolyze ATP or ADP to AMP. These enzymes can be involved in decreasing local host hemostasis when ADP is released by damaged cells and inhibits host platelet aggregation and inflammation [85]. Previous reports showed that 5'-nucleotidase/apyrase proteins expression decrease significantly after the blood meal, underscoring the important role of these proteins in the tick feeding process [86]. Similarly, in the soft tick *Ornithodoros savignyi*, apyrases were demonstrated to disaggregate platelets, confirming this protein family’s role in avoiding host platelet aggregation [87].

### Protease inhibitors

**Fig. 5:**
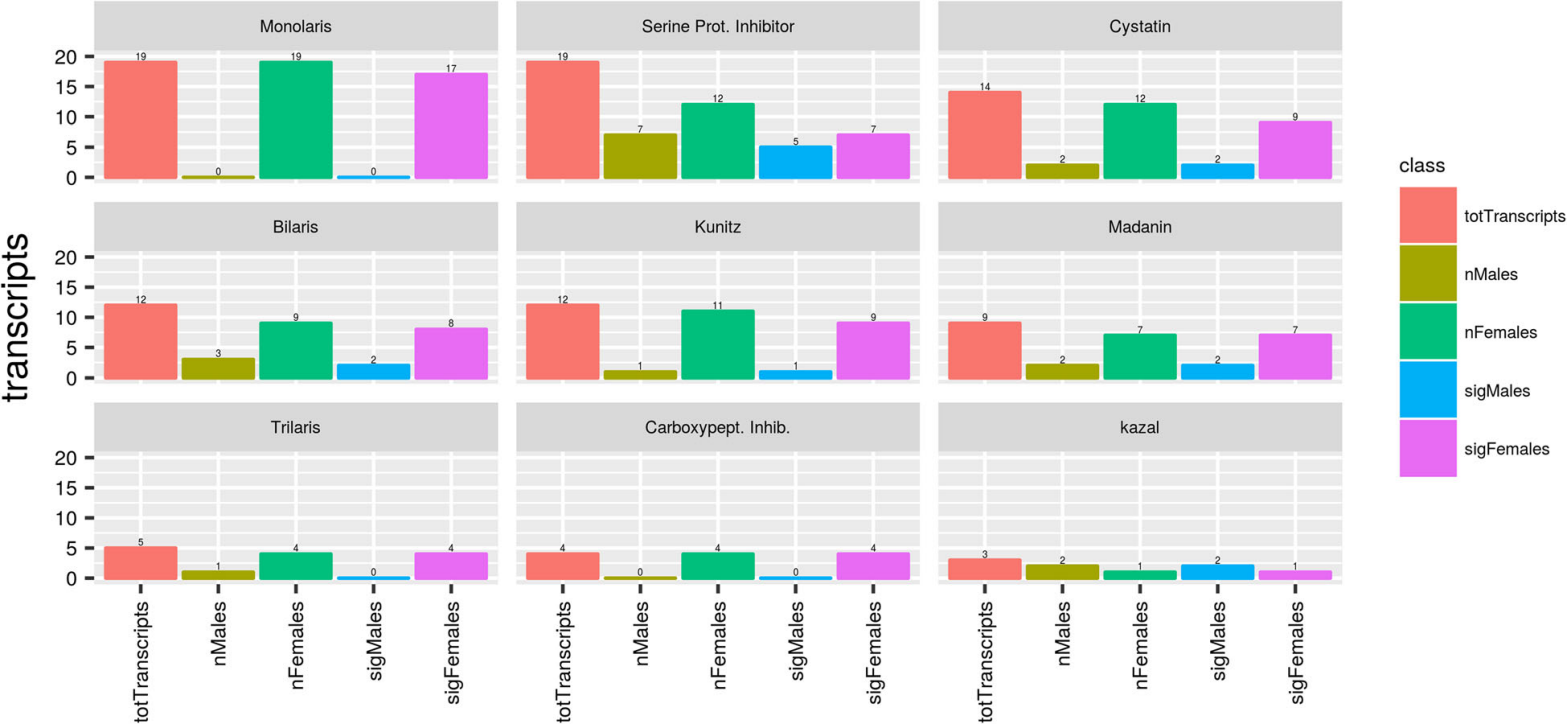
Differentially expressed protease inhibitor families in *H. dromedarii* male and female ticks

Protease inhibitors are abundantly expressed in tick sialotranscriptomes as members of a large gene family. In *H. dromedarii*, 100 transcripts (5.72% of secreted category) were associated with genes encoding for this protein family (Fig. 5). More details are shown below.

#### Kunitz domain-containing proteins

The Kunitz domain is 50–60 amino acid residues and its fold is highly conserved, resembling the first Kunitz-domain protein, the bovine pancreatic trypsin inhibitor (BPTI).

**Fig. 6:**
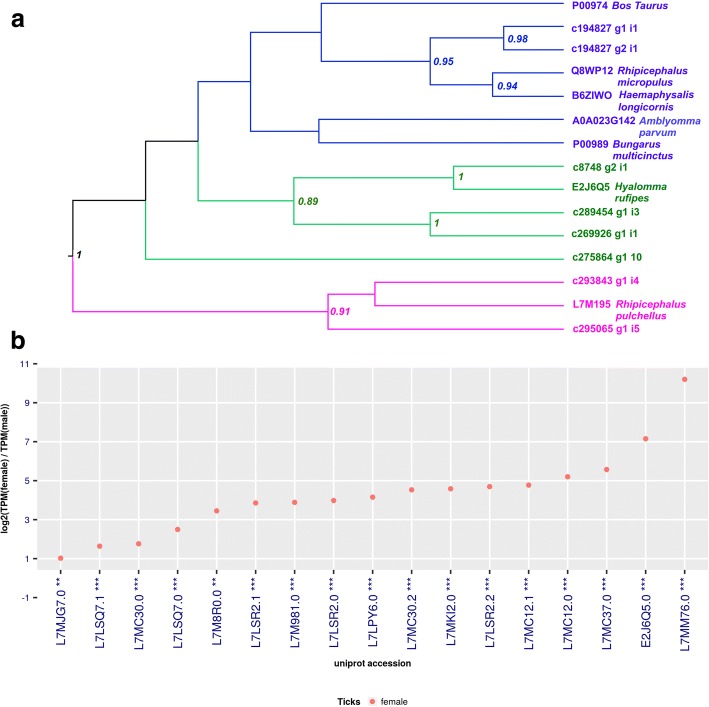
**a** Bayesian phylogenetic analysis of putative monolaris based on the Kunitz domain. The sequences from *Hyalomma dromedarii* were obtained in this study and sequences from other ticks species and vertebrates are indicated and referred to their GenBank accession numbers. **b** LFC between genders, for Monolaris, only female significant transcripts were found

Apart from the serine protease inhibitor function, Kunitz-domain proteins can also inhibit ion channels [88]. Kunitz-domain transcripts are one of the most abundant protein families in tick SGs. They are sub-classified by the number of Kunitz domains in each sequence (i.e. Monolaris, Bilaris, Trilaris). Interestingly, our study shows that 48 transcripts were related to Kunitz domain-containing proteins, of which 17 were Monolaris that were overexpressed exclusively in females (Fig. 6b). These proteins were described as having an anti-thrombin and an anti-factor Xa activity [89, 90]. Several Monolaris proteins were described in ticks, such as tryptogalinin, which may facilitate tick blood-feeding given that it inhibits several serine proteases involved in inflammation and vertebrate immunity [91]. Eight contigs of 17 were chosen for phylogenetic analysis, based on significant amino acid sequence difference, complete domain sequence and high expression values. We distinguished three major groups that share similarities (Fig. 6a). The clade indicated in blue is the most representative and the proteins assigned are mostly multifunctional Kunitz-type inhibitors acting mainly as coagulation enzymes, suggesting that they play an important role in maintaining blood fluidity during feeding of these parasites [92, 93]. The clade indicated in pink contains four contigs of putative Monolaris from *H. dromedarii* that seems to be exclusive from the genus *Hyalomma*, and is represented by a potential BPTI-Kunitz (E2J6Q5) [19]. The clade indicated in green contains three contigs of Monolaris from *H. dromedarii* and a putative Monolaris from *Rhipicephalus pulchellus.* Apparently, it does not contain molecules that clearly function to inhibit hemostatic processes already described. For a better understanding of the phylogenetic relationships of this family, it would be necessary to carry out further investigations for more species of high throughput data.

Our analysis shows that Bilaris, another Kunitz-family proteins, were overexpressed in *H. dromedarii* female ticks as compared to male ticks (8 *vs* 2 transcripts). Previous studies showed that three Bilaris proteins (Monobin, Ornithodorin and Savignin) have been characterized in the salivary glands from the soft ticks *A. monolakensis*, *O. moubata* and *O. savignyi*, respectively, and all were thrombin inhibitors [81, 86, 87, 94, 95].

#### Cystatins

In *H. dromedarii* sialotranscriptome, 14 transcripts were identified with 9 overexpressed in female and two in males (Fig. 5). This is the largest number of transcripts expressed in a tick species to date [22, 96]. The overexpression in males was attributed to the supposed role played by cystatins in reproduction as they are abundant in seminal fluid [21] but there is no evidence on the targets of the different types of cystatins or their involvement in blood-feeding or other processes. These cysteine protease inhibitors have been found previously in both hard and soft tick sialotranscriptomes and have been detected in several tick tissues [57, 97]. The family comprises large reversible and tight-binding inhibitors of papain-like enzymes and legumain [98]. There are four cystatin subgroups: type 1 (stefins), type 2, type 3 (kininogens) and type 4 cystatins (fetuins) [99]. Tick cystatins are either secreted as immunomodulators into the host with saliva or regulate hemoglobin digestion, which is driven by cathepsins [100]. Most tick cystatin transcripts are conserved across tick species and belong to the extracellular group, which suggests that their role is predominantly immunomodulatory [101].

#### Madanin

We annotated nine transcripts from *H. dromedarii* sialotranscriptome, seven of which were highly expressed in female *versus* two in male. Madanin, isolated for the first time, from the tick *Hae. longicornis* salivary glands, has an antithrombin activity [102]. This protein family was identified in the sialomes of other *Hyalomma* species such as *H. excavatum* and *H. rufipes* [19, 23]. In addition, variegin and chimadanin were isolated from *A. variegatum* and *Hae. longicornis*, respectively, as antithrombin peptides that act directly to inhibit blood clotting, making them very important for the blood-feeding process [103, 104].

#### Serine protease inhibitors

We identified 19 serpins in the *H. dromedarii* SG transcriptome of which five were significantly overexpressed in male *H. dromedarii* ticks and seven in females (Fig. 5). These different levels of expression could be partially explained by the ability of *H. dromedarii* males to feed on several hosts, require serpins expression that are different from the *H. dromedarii* female ticks [24]. Due to their abundance in the secretions of several organisms, serine protease inhibitors are the best-characterized family of protease inhibitors [105–107] and able to counterbalance host response to injury by inhibiting clotting and chymase [108, 109].

### Lipocalin

**Fig. 7:**
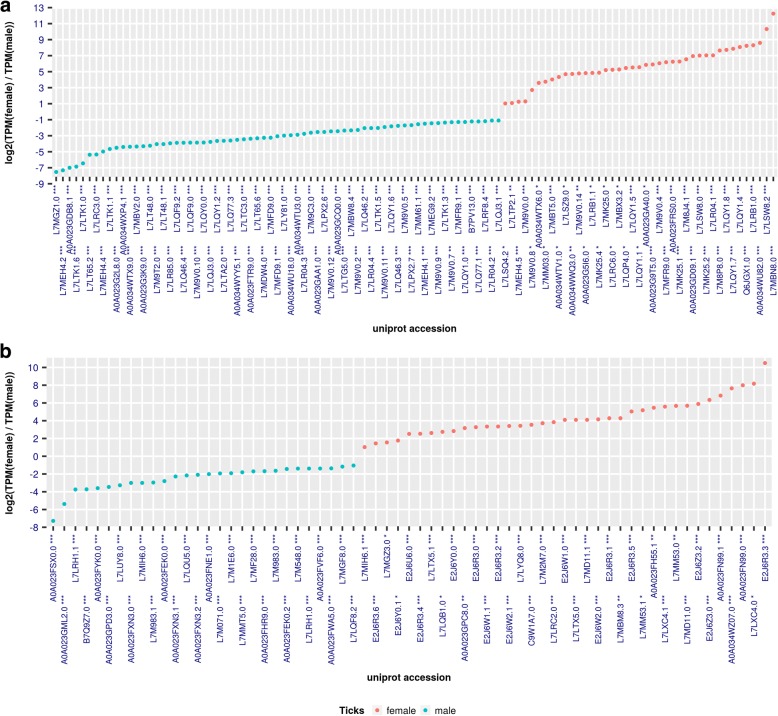
Calculated LFC = log2 (TPMfemale / TPM male) of H. dromedarri SG transcripts for different families. **a** Lipocalin. **b** Glycine-rich. Only transcripts with absolute value of LFC greater equal to 1 are plotted

Our analysis of *H. dromedarii* sialotranscriptome showed that 228 (13.04% of secreted class) transcripts were assigned to the lipocalin family, of which 139 were overexpressed in females and 89 in male *H. dromedarii* (Fig. 7b). Our results corroborate previously published data as lipocalins were found in almost all other tick sialomes for both genders [18, 19, 21, 74]. The widely spread lipocalin family is abundantly expressed in ticks and triatomine insects sialotranscriptomes and belong to a diverse gene family [57]. They are a family of small proteins (∼20 kDa) characterized by an eight-stranded antiparallel β-barrel fold with a repeated +1 topology, typically preceded by a short N-terminal 3_10_-helix and followed by a C-terminal α-helix. They frequently have one or more binding pocket(s) for small molecule ligand(s) [110]. In ticks, lipocalins were assigned to control inflammatory processes and interference with host homeostatic functions [63]. They were also found in nymph and adult tick saliva and are upregulated in response to injury and to viral or bacterial infections [111, 112].

### Glycine-rich

The present study demonstrated that the sialotranscriptome from *H. dromedarii* presents 101 transcripts related to glycine-rich proteins (Fig. 7a). A total of 68 transcripts were overexpressed in *H. dromedarii* female, while only 61, with statistically significant LFC, were overexpressed in male (Fig. 7b). The relative abundance of glycine was higher in female *H. dromedarii* ticks (41.63%) than in males (11.39%). This high abundance may reflect the fact that female *H. dromedarii* remain on the host for longer periods, making them more sensitive to removal. They therefore may secrete a cement in response to environmental threats such as host scratching. Glycine-rich proteins containing more than 60% glycine have been found in different tissues from many eukaryotic species [113] and in ticks, glycine-rich with other proteins constitute the cement that enables them to attach to their host [114]. These proteins, which resemble spider silk proteins, have been used as anti-tick vaccines [107–109].

### Mucin

Mucins are heavily glycosylated proteins with numerous functions including lubrication, cell signaling and host defense against pathogens [115]. Thirty transcripts of mucin were identified in *H. dromedarii* sialotranscriptome, which is higher than the number recorded in *H. excavatum* and lower than in *Rhipicephalus pulchellus*, which expresses 118 mucin-coding sequences [21, 96]. The involvement of mucins in the feeding process is not well elucidated making it difficult to explain the diversity of encoded mucins. They may function in tick feeding by coating the chitinous feeding mouthparts or the feeding lesion [62].

### Metastriate-specific

Proteins belonging to this family are specific to metastriatie ticks; no similar proteins exist in other arthropods. Thirty-five families of proteins totaling 196 sequences were found exclusively in metastriate arthropod genera. We found 132 transcripts related to metastriate-specific proteins, and discuss their sub-families below.

#### Evasin

Evasins are chemokine-binding proteins (CKBP) that differ from other CKBPs and whose molecular masses range from 7–11 kDa. In this study, 38 transcripts related to evasins were expressed in *H. dromedarii* SG. Their presence probably reflects the role in the inhibition of the recruitment of immune cells and therefore in reducing the risks that the host rejects the tick. Evasins are expressed more in *H. dromedarii* SG than in *H. excavatum* and *Rhipicephalus pulchellus* but less than in *Rh. appendiculatus* (34, 22 and 72, respectively). They were isolated from *Rh. sanguineus* for the first time and were also found in other metastriate sialotranscriptomes [74, 116].

#### Ixodegrin

Ixodegrin is a cysteine-rich family of proteins that was identified firstly in *I. pacificus* and *I. scapularis*ticks [56, 117]. This protein family has a predicted RGD or lysine, glycine, aspartic acid (KGD) domain indicative that interferes with fibrinogen binding to platelets [117]. Eighteen transcripts related to ixodegrin were assembled after reads sequencing and genes encoding for this family were expressed almost twice as much in male as in female ticks. As mentioned above, hemostasis starts within seconds of tissue injury and ticks face the launch of blood clotting cascades involving fibrinogen and leading to the platelet plug formation [118]. The expression of this family of proteins would therefore be expected for both genders of *H. dromedarii*; ixodegrin-like molecules are likely to be present in the saliva to inhibit the formation of platelet clot so as to facilitate the hematophagous feeding of fluid blood.

#### Da-p36

Our analysis showed that 10 transcripts were exclusively expressed in females of H. *dromedarii* compared to only one in males. DA-p36 was isolated for the first time from *Dermacentor andersoni* and it is a 36 kDa immunosuppressive protein that was widely found in metastriate ticks [119]. The presence of this protein family almost exclusively in *H*. *dromadriii* ticks can be related to the fact that female ticks are more exposed to host immune system because of their long-term feeding process.

### Gene enrichment analysis

In the functional annotation of genes based on KAAS, we identified 121 Metabolic pathways with 2966 transcripts in the Transcriptome Assembly, corresponding to 1710 unique orthologs and 621 enzymes (Table 2). The identification and categorization of The GeneMerge uses a hypergeometric distribution and applies a Bonferroni correction for a more appropriate and significant identification of enriched pathways. The analysis was divided into two sets with a group of 557 differentially expressed genes in females and another group of 353 differentially expressed genes in males. These two sets served as a filter to identfy the most enriched pathways with *P*-value ≤ 1 × 10^-3^ and FDR ≤ 1%.

### Gene enrichment, KEGG pathway analysis and enzyme classification

**Fig. 8:**
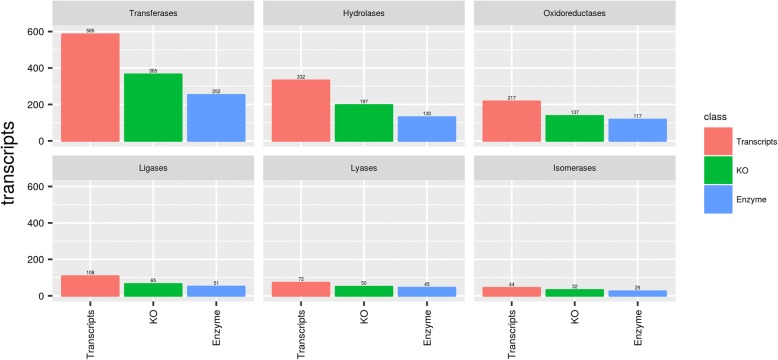
Enzyme Classification (EC) analysis of the transcriptome of *H dromedarii*. Number of EC number distribution of *H. dromedarii* compared with the number of transcripts, KEGG orthologs and enzymes

After the gene enrichment protocol, 7823 transcripts were identified exclusively in tick males and 4441 in tick females and 54,149 in both males and females. To evaluate the quality and coverage of *H. dromedarii* transcriptome assembly, we used the CEGMA pipeline to accurately annotate core genes [33], which showed that 248 (100%) of the Core Eukaryotic Genes (CEGs) were identified in the transcriptome and 241 (97.2%) of the CEGs were complete. Using the BUSCO core gene set which is based on orthologous genes from OrthoDBv9 [120], 937 (95.8%) proteins were identified from 978 core genes set, and only 41 conserved genes are missing (Table 3). In order to categorize and identify the biological pathways in *H. dromedarii*, the assembled contigs were used to obtain the Metabolic Pathways and Enzyme Commission (EC) when annotated against the KEGG database. A total of 2966 transcripts were assigned to 1710 unique KOs, 621 EC (Additional file 4: Table S4), and were summarized the number of specific and common transcripts in males and females in each enzyme classes (Additional file 5: Table S5). The ECs were subsequently grouped into 121 biochemical pathways. Only 4 pathways were not found based on 126 pathways from *Ixodes scapularis* genome (Glycosphingolipid biosynthesis-globo series, Glycosylphosphatidylinositol (GPI)-anchor biosynthesis, Mucin type O-Glycan biosynthesis, Regulation of autophagy). The enzyme sub-classes were distributed by the number of gender-specific and common transcripts, and the most representative sub-class “2.7 Transferring phosphorus-containing groups” has 224 classified transcripts and one of the most important enzyme sub-classes “3.4 Acting on peptide bonds (peptidases)” has 52 classified transcripts. After the assignment of KEGG pathways annotation to the assembled transcripts, the potential enzymes were further characterized using the predictions of Enzyme Commission (EC) numbers for each transcript (Additional file 5: Table S5). Enzyme classification revealed that transferases are the largest group of *H. dromedarii* enzymes (40.58%, 252 enzymes), followed by hydrolases (20.93%, 130 enzymes), oxidoreductases (18.84%, 117 enzymes), ligases (8.2%, 51 enzymes), lyases (7.2%, 45 enzymes) and isomerases (4%, 25 enzymes) (Fig. 8). The 1341 sequences having EC numbers were further characterized by the Kyoto Encyclopedia of Genes and Genomes (KEGG) pathway analysis. Interestingly, a large number of transcripts were found to be associated with Biosynthesis of antibiotics (197 transcripts), possibly indicating interesting genes for future drug target discovery studies.

**Table 3:** Identification of ultra-conserved eukaryotic proteins from BUSCO

Description	Total	% of total
Complete single-copy BUSCOs	525	53.7
Complete duplicated BUSCOs	393	40.2
Fragmented BUSCOs	19	1.9
Missing BUSCOs	41	4.2
Total BUSCO groups searched	978	–

## Conclusions

Transcript expression differed for male and female *H. dromedarii* ticks, which might be related to their feeding behaviors. The complexity and diversity of *H. dromedarii* transcriptome corroborated previous studies and may potentially reflect adaptation to the complexity of the host’s defense mechanisms. Our results contribute to the understanding of the tick-host molecules interaction during blood-feeding and the discovery of new pharmacologically active proteins of *Hyalomma* ticks. Our study has clearly enabled the creation of a database that will serve further proteomic and functional studies. The development of approaches to the identification of tick salivary proteins points the way to several directions in the areas of biomedical, veterinary and pharmacological work identifying vaccine targets that would disrupt the blood meal and/or the transmission of pathogens.

## Additional files

Additional file 1: **Table S1.** Annotated transcripts of *H. dromedarii* salivary glands. (XLSX 17611 kb)

Additional file 2: **Table S2.** Housekeeping class transcripts and their annotations. (XLSX 775 kb)

Additional file 3: **Table S3.**Transposable elements transcripts and their annotations. (XLS 2170 kb)

Additional file 4: **Table S4.** Distribution of putative transcripts in KEGG pathways. (XLSX 18 kb)

Additional file 5: **Table S5.** Enzyme Classification (EC) analysis of the transcriptome of *H. dromdarii*. Distribution of EC subClasses number in general EC terms compared to the number of specific transcripts in male, female and in common. (XLSX 10 kb)

## Abbreviations

ADAM: A disintegrin and metalloprotease
ADP: Adenosine diphosphate
AMP: Adenosine monophosphate
ATDB: Animal Toxin Database
ATP: Adenosine triphosphate
BBH: Bi-directional best hit
BPTI: Bovine pancreatic trypsin inhibitor
cDNA: complementary DNA
CEGs: Core eukaryotic genes
CKBP: Chemokine-binding proteins
dCAS: Desktop annotation system
EC: Enzyme Commission
FPKM: Fragments per kilobase of exon per million fragments mapped
GO: Gene Ontology
GPI: Glycosylphosphatidylinositol
KEGG: Kyoto Encyclopedia of Genes and Genomes
KGD: lysine, glycine, aspartic acid
KO: KEGG Orthology
KOG: Eukaryotic ortholog groups
LFC: Log fold change
MCMC: Markov chain Monte Carlo
MDC: Metalloproteinase/disintegrin/cysteine-rich
mRNA: Messenger RNA
NCBI: National Center for Biotechnology Information
NET: Neutrophils extracellular traps
NGS: Next-generation sequencing
NR: Non-redundant protein database
ORF: Open reading frame
PBS: Phosphate-buffered saline
RGD: Arginylglycylaspartic acid
RNAseq: RNA sequencing
rRNA: Ribosomal RNA
RT-PCR: Reverse transcription polymerase chain reaction
SAT: Saliva-assisted transmission
SG: Salivary glands
TEs: Transposable elements
TPM: Transcripts per million
